# Associations of dietary riboflavin intake with coronary heart disease in US adults: a cross-sectional study of NHANES 2007–2018

**DOI:** 10.3389/fnut.2024.1467889

**Published:** 2024-12-12

**Authors:** Qiqi Jin, Shanjiang Chen, Xiaojun Ji

**Affiliations:** Department of Cardiology, Wenzhou Central Hospital, Wenzhou, China

**Keywords:** riboflavin, coronary heart disease, CHD, National Health and Nutrition Examination, cross-sectional study

## Abstract

**Objective:**

There is currently little study on the relationship between dietary riboflavin intake and coronary heart disease (CHD) risk.

**Methods:**

Using information from the National Health and Nutrition Examination Survey (NHANES) between 2007 and 2018, we carried out a cross-sectional study. Dietary riboflavin intake and CHD risk were examined using weighted univariate and multivariable logistic regression. To learn more about the connection between dietary riboflavin intake and CHD risk, subgroup analyses and interactions were conducted. Next, the potential non-linear association was visually described using restricted cubic spline (RCS).

**Results:**

The risk of CHD was inversely correlated with dietary riboflavin consumption. The multivariable odds ratio (OR) with 95% confidence interval (CI) for the risk of CHD was 0.52 (95%Cl: 0.34–0.81, P_trend_ = 0.009) for the highest vs. lowest tertiles of riboflavin. This protective effect of dietary riboflavin on CHD was influenced by gender, drinking status and serum folate concentration. A non-linear inverse connection (P_for nonlinearity_ ≤ 0.001) was shown using RCS analysis between riboflavin intake and the risk of CHD.

**Conclusion:**

Our research suggested that consuming more riboflavin in your diet may lessen the risk of CHD. The results improved the current knowledge base and supplied potential implications for dietary recommendations and health policy.

## 1 Introduction

Coronary heart disease (CHD) is a chronic and intricate illness with a high morbidity and mortality (1). It is primarily caused by the development of fatty deposits in the coronary arteries, which can ultimately result in a myocardial infarction or stroke (2). From 1990 to 2019, there was an estimated increase in the global mortality rate of CHD from 106.47 per 100,000 to 118.10 per 100,000, with a proportion of deaths to total deaths rising from 12.21 to 16.17%, emerging CHD as a significant global health concern (3, 4). Given the serious consequences associated with CHD, it is imperative to ascertain efficacious non-pharmaceutical interventions that can effectively mitigate its incidence.

Diet plays a crucial role in the development and prevention of CHD, and various dietary elements, such as dietary omega-3 (3), inflammatory index (5), vitamin K (6), magnesium (7), L-arginine (8), and fiber (9), calcium (10), vitamin D (10), vitamin A (11) and pistachios (12) have been scientifically proven to be associated with the development of CHD. B vitamins, a set of water-soluble vitamins, are vital in the degradation of homocysteine (Hcy), and elevated Hcy level has been recognized as a standalone risk factor for CHD (13, 14). So, the deficiency of B vitamins might be link to the prevalence of CHD. However, most current research centered on the relationship of vitamin B6, vitamin B12, and folic acid with CHD, and few studies focused on the association between riboflavin and CHD (15, 16).

Riboflavin (vitamin B2), a member of vitamin B family, is present in several plant and animal foods such as enriched flour, meat, milk, eggs, nuts, and green vegetables (17). Humans lack the ability to synthesize riboflavin and must acquire it through food or supplementation (18). Riboflavin is a essential constituent of coenzymes that participate in the processes of cellular proliferation, energy production, and adipose tissue degradation (19). According to researches, riboflavin deficiency has been linked to the onset of several cardiovascular conditions outside CHD, including stroke (20, 21), myocardial infarction (22), and heart failure (23). Additionally, those who consumed more riboflavin had a reduced mortality rate from cardiovascular disease than those who consumed less (24). In order to investigate the connection of dietary riboflavin intake with CHD risk in adults, we conducted this study using a sizable sample from the National Health and Nutrition Examination Survey (NHANES).

## 2 Methods

### 2.1 Study population

The NHANES survey assessed the nutritional and health condition of the U.S. population by conducting interviews, physical examinations, and laboratory tests. It employed a sophisticated sampling design that is multilevel, stratified, and clustered, ensuring that the sample is highly representative of the overall population (25). The protocol implementations of the study were evaluated and approved by the ethics review committee of the National Center for Health Statistics (NCHS). Additionally, all participants in the study submitted informed permission forms (26). The study utilized openly accessible NHANES data and did not necessitate any supplementary ethical clearance or authorization (3).

**Figure 1:**
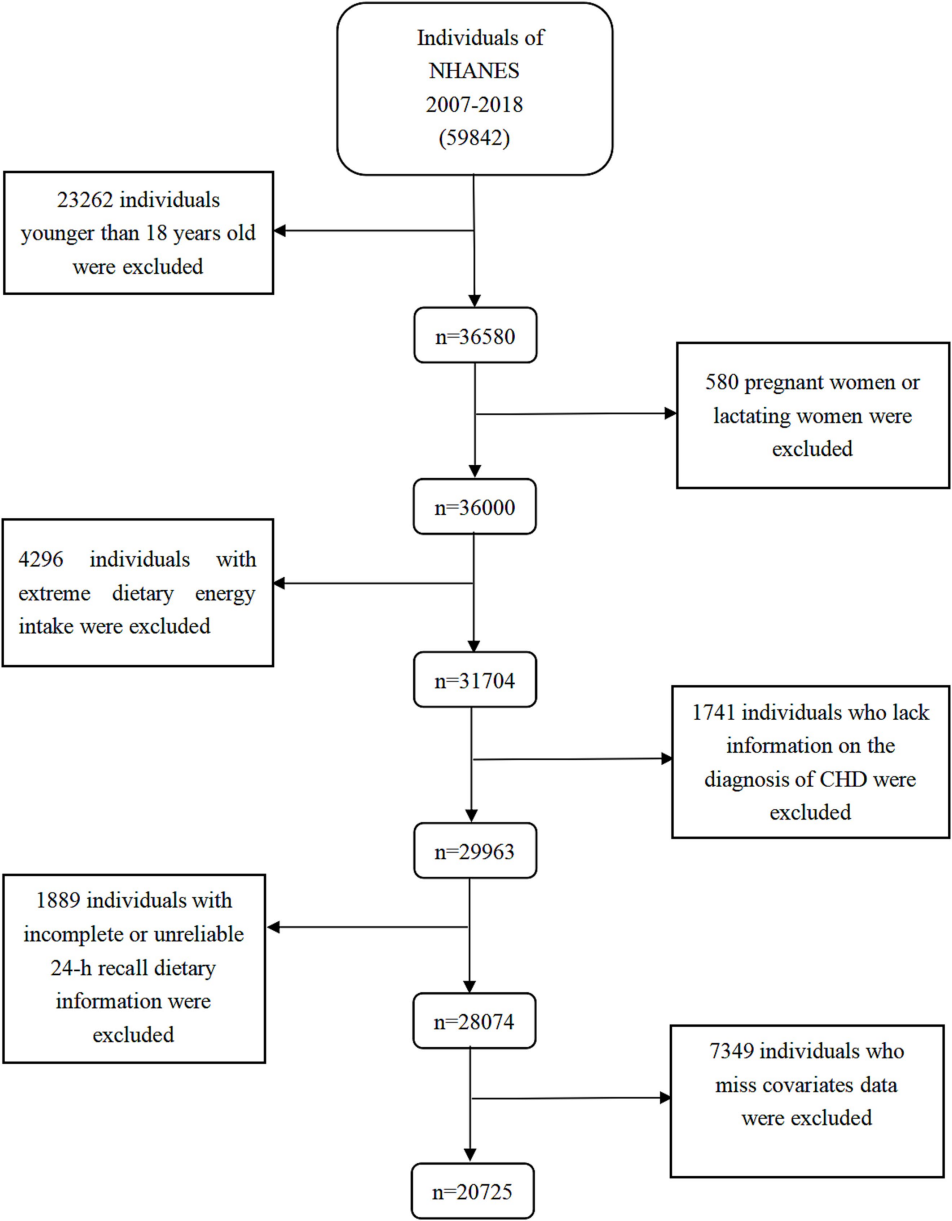
Flow chart of the screening process for the selection of eligible participants. NHANES, National Health and Nutrition Examination Survey.

This study combined data from six consecutive biennial cycles (2007–2018), encompassing a total of 59,842 participants. We gradually removed individuals under the age of 18 (*n* = 23,262), pregnant/nursing women (*n* = 580), those with extreme calorie intakes (*n* = 4,296), those lacking information on CHD diagnosis (*n* = 1,741), those with inaccurate or incomplete 24-h recall dietary data (*n* = 1,889), and those missing covariates variables data (*n* = 7,349). Finally, the study included a total of 20,725 individuals (10,156 men and 10,569 women). Figure 1 and Supplementary Table S1 illustrated the intricate procedure.

### 2.2 Self-reported history of CHD outcome

The diagnosis of CHD used in this study was based on the self-reports provided by the participants. In the health-related questionnaire, they were asked “Have you been told by a doctor or other health professional that you have CHD?” If an individual responds “yes,” it would indicate an adverse outcome for CHD.

### 2.3 Assessment of dietary riboflavin intake

Diet-related questionnaires were used to measure the amount of riboflavin consumed through diet. The automated multiple-pass method (AMPM) used in these questionnaires allowed for a potential discrepancy in the acquired nutritional intake data from the actual intake of within 10% (27). For the first time in an interview, participants were asked to recollect what they had eaten and drunk over the previous 24 h. To set up the second visit, a phone call was placed between days three and ten. The Food Intake Analysis System (FIAS) and United States Department of Agriculture (USDA) survey nutrient database were utilized to encode and transform dietary data into total nutrient intake, and minimize the differences in participants’ dietary riboflavin intake. The 24-h dietary supplement usage component was performed following the 24-h dietary survey for food and beverage consumption. The average intake from two 24-h food surveys and two 24-h supplement surveys was used to calculate the dietary riboflavin consumption.

### 2.4 Covariates

In this study on dietary factors and cardiovascular disease, several variables were included as covariates to mitigate potential confounding effects, refer to previous studies (28, 29). Some socioeconomic status variable (such as age), health status variables (such as hypertension), and health behavior variables (such as drinking status), were incorporated. We also adjusted for the overall intake of energy. Additionally, considering the interaction between folate and riboflavin during metabolism, we further adjusted serum folate concentration (30). Supplementary Table S2 presented comprehensive categorizations of all covariates.

### 2.5 Statistical analysis

In this study, we employed R software (version 4.3.2) and SPSS version 27.0 for the main statistical analysis. New sample weights were created by integrating continuous data from 62-year cycles, in accordance with the NHANES analysis criteria (31).

Categorical variables were shown as number (n) and percentages (%), and continuous variables were represented in the baseline data by means and standard deviation (SD). Then, to evaluate the variations in individual characteristics between the groups with and without CHD outcomes, the student’s t-test and Chi-square test were used.

The weighted logistic regression models were used to investigate the correlations between dietary riboflavin intake and the risk of CHD. In models, the subjects were divided into three equally spaced groups: Tertile 1 (<1.65 mg/day), Tertile 2 (1.65 to 2.78 mg/day), and Tertile 3 (>2.78 mg/day) based on he distribution of riboflavin intake, dietary riboflavin intake was treated as the independent variable, and stroke event was treated as the dependent variable. Model 1 adjusted for age and sex, Model 2 adjusted for all factors in Supplementary Table S1 with the exception of serum folate concentration, and Model 3 incorporated serum folate concentration additionally. Subgroup analyses and interactions were carried out to investigate the stability of the outcomes. To explore dose–response relationship, dietary riboflavin intake was examined as a continuous variable, and restricted cubic splines (RCS) model with 3 knots (at the 5th, 50th, and 95th percentiles) was used to handle the non-linear correlations between stroke event and dietary riboflavin intake. The study’s findings were statistically significant when the two-sided *p* value was less than 0.05.

## 3 Results

### 3.1 Basic characteristics of all participants

20,725 individuals in all made up the research. Of them, 9,404 (45.38%) were Non-Hispanic White, 4,158 (20.06%) were Non-Hispanic Black, 2,094 (10.10%) were other race, and 3,011 (14.53%) were Mexican Americans. The participants’ average age was 49.65 ± 17.47 years. Of these 20,725, 850 (4.10%) had a CHD incident. The distribution characteristics of the groups with and without CHD result were compared, and the results were shown in Table 1. The individuals included in the CHD cases were found to be older, primarily male, non-smokers, obese, married or cohabiting, drinkers, and had higher rates of hypercholesterolemia and hypertension. Additionally, it was clear that CHD patients demonstrated a lower intake of riboflavin and higher level of serum folate concentration than non-CHD patients.

**Table 1:** Characteristics of participants by CHD status, NHANES 2007–2018.

Characteristics	Overall	CHD	Non-CHD	*P* value
Number of subjects (%)	20,725 (100.00)	850 (100.00)	19,875 (100.00)	
Gender (%)^1^				< 0.001
Male	10,156 (49.00)	584 (68.71)	9,572 (48.16)	
Female	10,569 (51.00)	266 (31.29)	10,303 (51.84)	
Race (%)^1^				< 0.001
Mexican American	3,011 (14.53)	71 (8.35)	2,940 (14.79)	
Other Hispanic	2058 (9.93)	68 (8.00)	1990 (10.01)	
Non-Hispanic White	9,404 (45.38)	554 (65.18)	8,850 (44.53)	
Non-Hispanic Black	4,158 (20.06)	105 (12.35)	4,053 (20.39)	
Other Race	2094 (10.10)	52 (6.12)	2042 (10.27)	
Age (%)^1^				< 0.001
18–39	6,790 (32.76)	14 (1.65)	6,776 (34.09)	
40–59	7,010 (33.82)	134 (15.76)	6,876 (34.60)	
≥60	6,925 (33.41)	702 (82.59)	6,223 (31.31)	
Body mass index (%)^1^				< 0.001
<25 kg/m^2^	5,851 (28.23)	182 (21.41)	5,669 (28.52)	
25–30 kg/m^2^	6,807 (32.84)	289 (34.00)	6,518 (32.79)	
≥30 kg/m^2^	8,067 (38.92)	379 (44.59)	7,688 (38.68)	
Educational level (%)^1^				< 0.001
<High school	1810 (8.73)	114 (13.41)	1,696 (8.53)	
High school	7,500 (36.19)	345 (40.59)	7,155 (36.00)	
>High school	11,415 (55.08)	391 (46.00)	11,024 (55.47)	
Smoking status (%)^1^				< 0.001
Yes	5,190 (25.04)	387 (45.53)	4,803 (24.17)	
No	15,535 (74.96)	463 (54.47)	15,072 (75.83)	
Drinking status (%)^1^				0.964
Yes	14,059 (67.84)	576 (67.76)	13,483 (67.84)	
No	6,666 (32.16)	274 (32.24)	6,392 (32.16)	
Poverty-income ratio (%)^1^				0.219
<1.00	4,272 (20.61)	161 (18.94)	4,111 (20.68)	
≥ 1.00	16,453 (79.39)	689 (81.06)	15,764 (79.32)	
Recreational activity (%)^1^				< 0.001
Vigorous	4,683 (22.60)	64 (7.53)	4,619 (23.24)	
Moderate	5,532 (26.69)	249 (29.29)	5,283 (26.58)	
Other	10,510 (50.71)	537 (63.18)	9,973 (50.18)	
Work activity (%)^1^				< 0.001
Vigorous	4,192 (20.23)	116 (13.65)	4,076 (20.51)	
Moderate	4,564 (22.02)	191(22.47)	4,373 (22.00)	
Other	11,969 (57.75)	543 (63.88)	11,426 (57.49)	
Sleeping disorder (%)^1^				< 0.001
Yes	637 (3.07)	268 (31.53)	369 (1.86)	
No	20,088 (96.93)	582 (68.47)	19,506 (98.14)	
Marital Status (%)^1^				< 0.001
Married/Living with partner	12,424 (59.95)	532 (62.59)	11,892 (59.83)	
Widowed/Divorced/Separated	4,554 (21.97)	279 (32.82)	4,275 (21.51)	
Never married	3,747 (18.08)	39 (4.59)	3,708 (18.66)	
Diabetes status(%)^1^				< 0.001
Yes	3,670 (17.71)	362 (42.59)	3,308 (16.64)	
No	17,055 (82.29)	488 (57.41)	16,567 (83.36)	
Hypertension status(%)^1^				< 0.001
Yes	11,307 (54.56)	718 (84.47)	10,589 (53.28)	
No	9,418 (45.44)	132 (15.53)	9,286 (46.72)	
Hypercholesterolemia status(%)^1^				< 0.001
Yes	7,579 (36.57)	690 (81.18)	6,889 (34.66)	
No	13,146 (63.43)	160 (18.82)	12,986 (65.34)	
Below riboflavin RDA (%)^1^	3,367 (16.25)	131 (15.41)	3,236 (16.28)	0.535
Dietary riboflavin intake (mg/day)^2^	3.92 ± 9.75	3.73 ± 6.81	3.93 ± 9.85	0.039
Total energy intake (kcal/d)^2^	2047.83 ± 815.54	1884.86 ± 698.31	2054.80 ± 819.48	< 0.001
Serum folate concentration (nmol/l)^2^	43.58 ± 32.19	54.28 ± 38.12	43.13 ± 31.83	< 0.001

CHD, coronary heart disease; NHANES, National Health and Nutrition Examination Survey.

1 Chi-square test was used to compare the percentage between participants with and without CHD.

2 Student’s *t*-test was used to compare the mean values between participants with and without CHD.

### 3.2 Association between dietary riboflavin intake and the risk of CHD

Table 2 presented the findings of the logistic regression analysis conducted to examine the relationship between dietary riboflavin intake and CHD risk. In the crude model, dietary riboflavin intake was not correlated with the risk of CHD. In Model 1, a higher dietary riboflavin intake was associated with a reduced risk of CHD. People who consumed more riboflavin in their diet (Tertile 3) had a 41% lower incidence of CHD than those who consumed less (Tertile 1). The associated odds ratio (OR) with 95% confidence interval (CI) was 0.59 (0.45–0.76) for Tertile 3. Model 2 continued to identify a negative correlation between riboflavin consumption and the risk of CHD. In contrast to Tertile 1, those who consumed riboflavin through their diets in Tertile 3 had an OR (95%CI) of 0.53 (0.38–0.83) for CHD risk. Model 3 further reinforced the inverse correlation between dietary riboflavin and CHD risk by adjusting for serum folate levels. Compared to low level (Tertile 1) of riboflavin intake, the OR (95% CI) for high level (Tertile 3) was 0.52 (0.34–0.81) for CHD risk.

**Table 2:** Weighted ORs (95%CIs) for CHD according to tertiles of dietary riboflavin intake, NHANES 2007–2018.

Intake cutoff		Cases/participants^1^	Crude^2^	Model 1^2^	Model 2^2^	Model 3^2^
			OR (95%CI)	OR (95%CI)	OR (95%CI)	OR (95%CI)
Dietary riboflavin intake (mg/day)						
Tertile 1 (low)	< 1.65	255/6908	1 (ref)	1 (ref)	1 (ref)	1 (ref)
Tertile 2	1.65–2.78	294/6909	1.00 (0.77–1.31)	0.79 (0.58–1.06)	0.76 (0.53–1.09)	0.74 (0.51–1.07)
Tertile 3 (high)	≥ 2.78	301/6908	0.99 (0.77–1.28)	0.59 (0.45–0.76)^**^	0.53 (0.38–0.83)^**^	0.52 (0.34–0.81)^**^
*p*_trend_			<0.001	<0.001	0.001	0.009

OR, odd ratio; CI, confidence interval; CHD, coronary heart disease; NHANES, National Health and Nutrition Examination Survey. Model 1 adjusted for age and gender. Model 2 adjusted for gender, race, age, body mass index, educational level, marital status, smoking status, poverty-income ratio, sleeping disorder, drinking status, hypertension status, work activity, recreational activity, hypercholesterolemia status, diabetes status, and dietary energy intake. Model 3 added serum folate concentration on the basis of model 2. Results were dietary-weighted. *p<0.05; **p<0.01.

1 Cases of CHD/number of participants in tertiles.

2 Calculated using binary logistic regression.

### 3.3 The results of subgroup analyses and interactions

**Figure 2:**
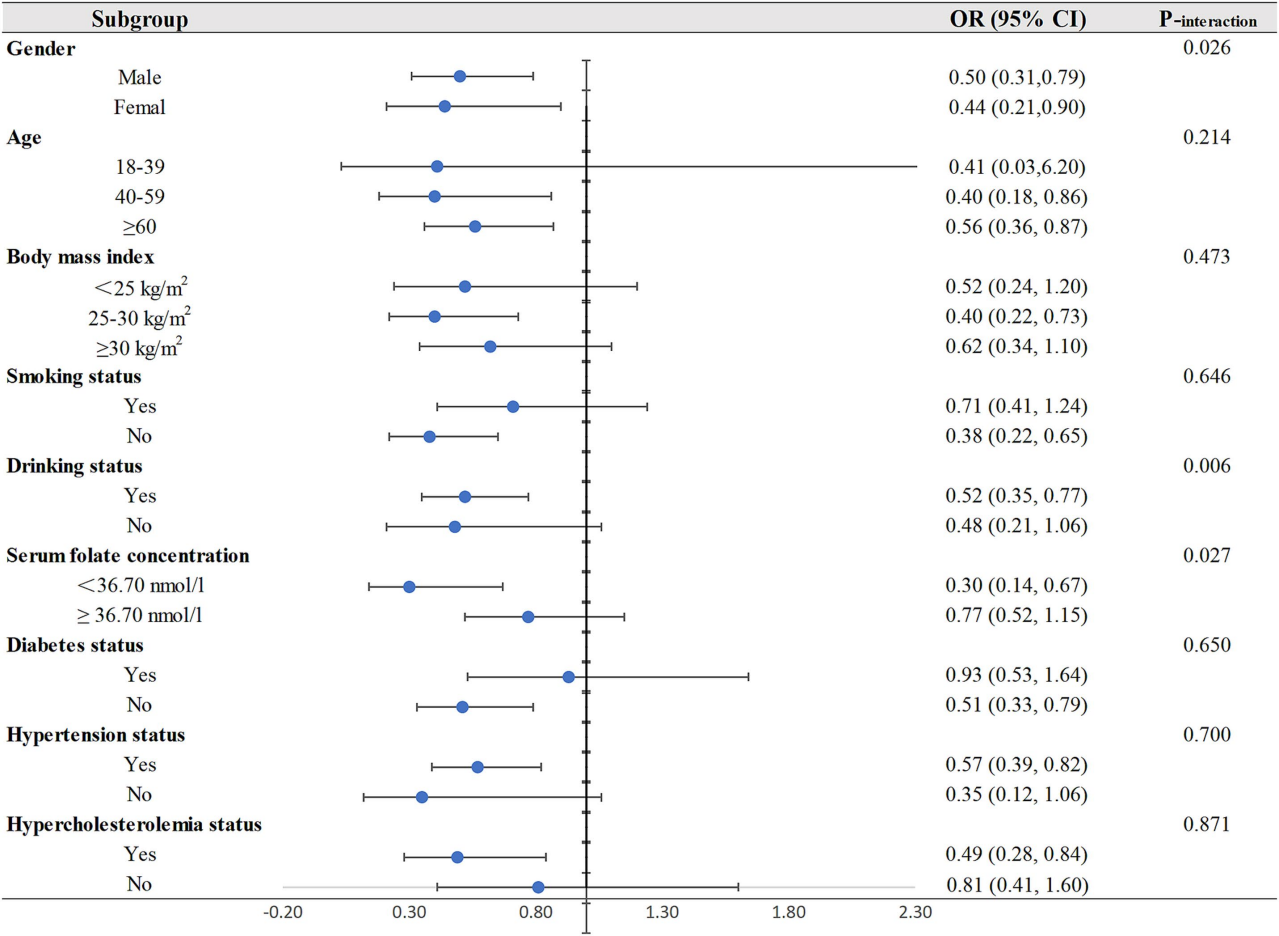
Subgroup analyses for the relationship between dietary riboflavin intake and CHD. OR, odd ratio; CI, confidence interval; CHD: coronary heart disease.

Subsequently, we performed the subgroup analyses and interactions, and the resulting *p* values and ORs (95% CIs) were visually represented through forest plots in Figure 2. The protective effect of dietary riboflavin intake on CHD risk was not influenced by age, body mass index, smoking or not, and the status of hypertension, diabetes and hypercholesterolemia. The inverse correlation of riboflavin with CHD was more significant for older individuals, participants with body mass index between 25 and 30, non-smokers, participants with hypertension or hypercholesterolemia and participants without diabetes.

The results of interactions shown that there was a significant interaction of dietary riboflavin intake with gender (P_interaction_ = 0.026), drinking status (P_interaction_ = 0.006), and serum folate concentration (P_interaction_ = 0.027). In the subgroup analysis by gender, dietary riboflavin was negatively associated with CHD risk in both men and women, and this inverse association was only observed in drinkers in the subgroup analysis of drinking status. In the subgroup analysis of serum folate concentration, we found a protective effect of dietary riboflavin on CHD risk only in people with low serum folate levels but not in people with high serum folate levels.

### 3.4 Nonlinear association

**Figure 3:**
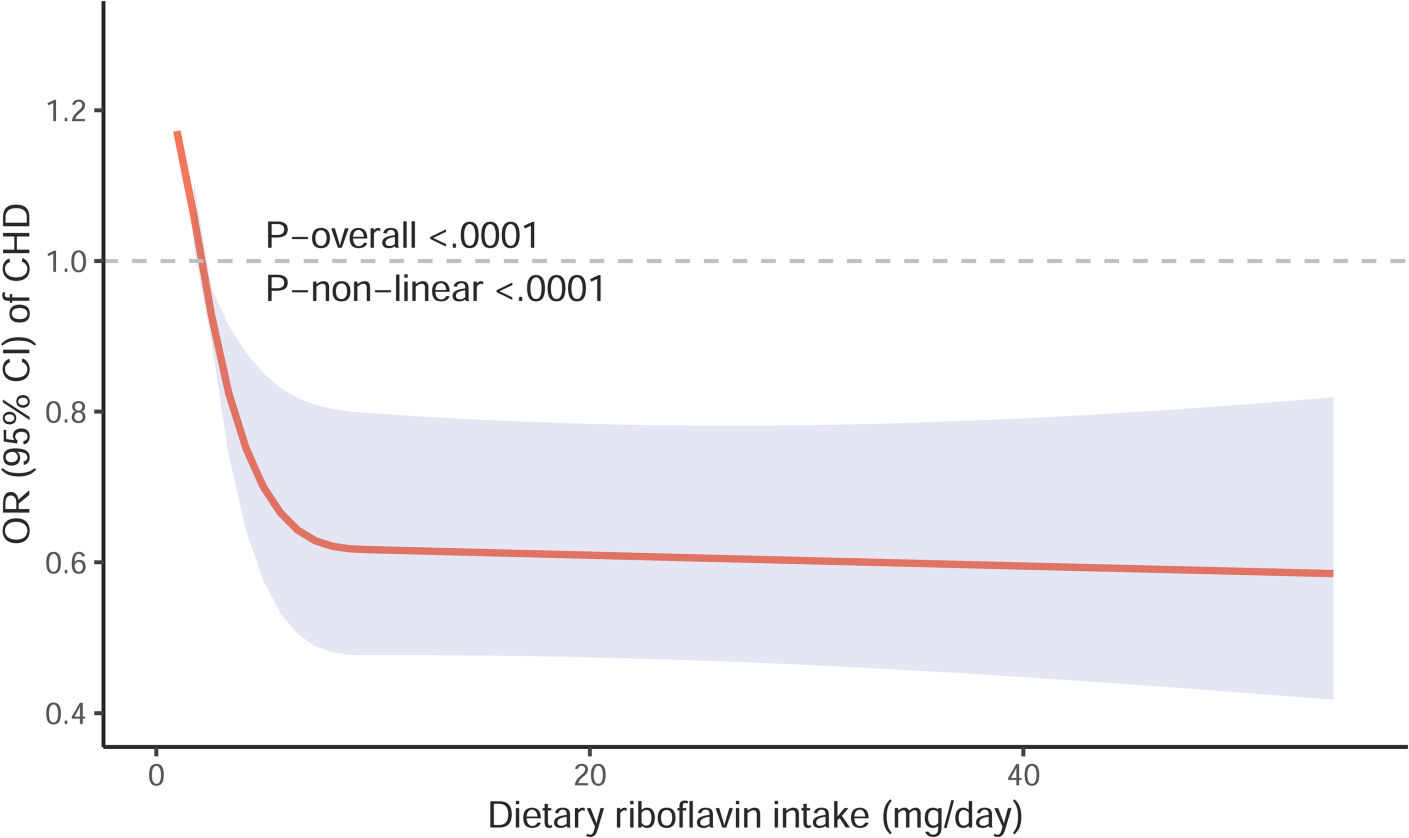
Restricted cubic spline models for the relationship between Restricted cubic spline models for the relationship between dietary riboflavin intake and the risk of CHD. The 95% CIs of the adjusted ORs are represented by the gray-shaded area. The model is adjusted for gender, race, age, body mass index, educational level, marital status, smoking status, poverty-income ratio, sleeping disorder, drinking status, hypertension status, work activity, recreational activity, hypercholesterolemia status, diabetes status, dietary energy intake and serum folate concentration. OR, odd ratio; CI, confidence interval; CHD, coronary heart disease.

A clear L-shaped dose–response connection was seen between the intake of riboflavin in the diet and the risk of CHD, as indicated by a *p* value of ≤0.001 for non-linearity (Figure 3). This indicated that an increase in dietary riboflavin intake was associated with a considerable decrease in the risk of CHD when the daily intake was below 10.50 mg, while the intake reached 10.50 mg/day, the risk of CHD remained relatively stable.

## 4 Discussion

This was the first study, as far as we know, to comprehensively investigate the associations between dietary riboflavin intake and CHD risk using NHANES data, filling a gap in the existing literature on riboflavin and CHD. The prevalence of CHD was 4.10% in this study. The results of multivariate logistic regression revealed a correlation between a decreased risk of CHD and a higher dietary riboflavin intake. Subgroup analyses results indicated that the negative relationship between dietary riboflavin intake and the risk of CHD may be influenced by gender, drinking status, and serum folate concentration (P_interaction_ ≤ 0.05). Additionally, a non-linear inverse association between riboflavin intake and the risk of CHD was indicated by RCS curves.

**Figure 4:**
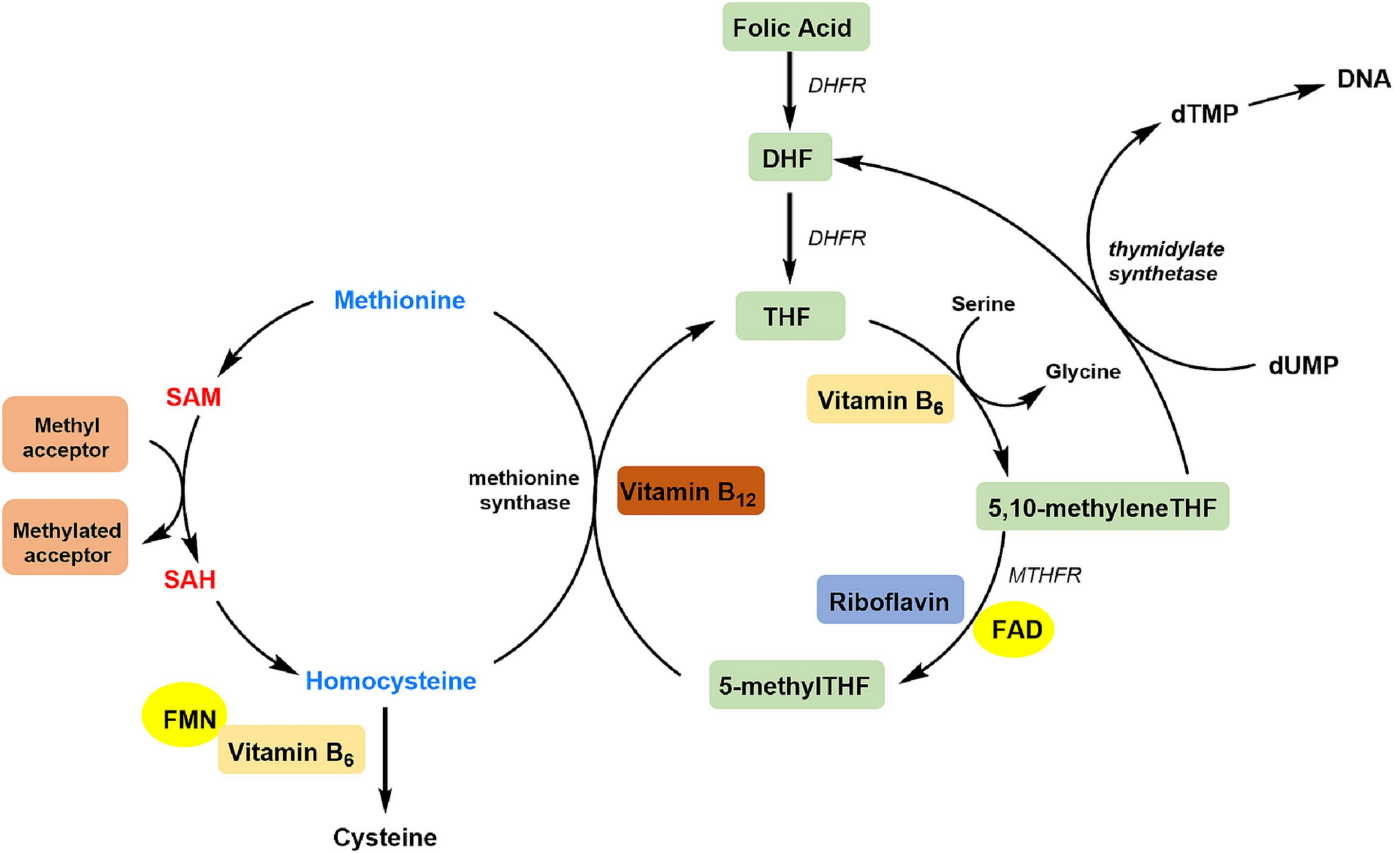
Overview of riboflavin in the C1 cycle. DHF, dihydrofolate; DHFR, dihydrofolate reductase; THF, tetrahydrofolate; dTMP, deoxythymidine monophosphate; dUMP, deoxyuridine monophosphate; MTHFR, methylenetetrahydrofolate reductase; FAD, flavin adenine dinucleotide; FMN, flavin mononucleotide; SAH, S-adenosylhomocysteine; SAM, S-adenosylmethionine.

The protective effect of riboflavin on CHD may be due to its involvement in C1 metabolism by its two active cofactor forms, flavin adenine dinucleotide (FAD) and flavin mononucleotide (FMN) (Figure 4) (32). FAD is a co-factor for methylenetetrahydrofolate reductase (MTHFR), which facilitates the conversion of 5,10-methylenetetrahydrofolate to 5-methyltetrahydrofolate. 5-Methyltetrahydrofolate donates a methyl group, allowing Hcy to be converted back into methionine (33, 34). FMN is needed to produce pyridoxal phosphate (the active coenzyme form of vitamin B6), which serves as a cofactor of cystathionine *β*-synthase and other trans-sulfuration enzymes (32). Riboflavin shortage may cause a rise in Hcy levels, which is a independent predictor of cardiovascular disease (35).

In the folate C1 metabolic cycle, riboflavin is frequently disregarded in favor of folate, vitamin B12, and vitamin B6, but it is essential for those who have the homozygous mutant 677TT genotype in MTHFR, which affects roughly 10% of persons globally (36). A lower affinity for FAD cofactors is caused by the 677C → T variation in the MTHFR gene, which lowers enzyme activity (37). According to statistics, those with the MTHFR 677TT genotype exhibit low folate concentrations and a high plasma Hcy level, as well as a 70% decrease in MTHFR activity when compared to those with the MTHFR 677CC genotype (38–40). A higher riboflavin status can stabilize the variant form of the enzyme by either preventing the FAD cofactor from exiting the active site or facilitating its prompt replacement, particularly in individuals with low folate levels (41). This just clarified the interaction of serum folate concentration on the inverse association of dietary riboflavin with CHD found in our study, and the protective effect of dietary riboflavin on CHD was more pronounced in the group with low serum folate level. we also found an interactive effect of drinking status on the association between dietary riboflavin and CHD, possibly because alcohol consumption affects riboflavin absorption and exacerbates riboflavin deficiency (24, 42). Gender had an effect of riboflavin intake on CHD risk may be due to differences in hormone levels between men and women.

In this study, the mean dietary riboflavin intake of participants was found to exceed the Recommended Dietary Allowance (RDA) recommendations of 1.30 mg/day for adult men and 1.10 mg/day for adult women (43). According to the dose–response results, the risk of coronary heart disease gradually decreased with the increase of riboflavin intake, and the protective effect of riboflavin against coronary heart disease became statistically significant in the range of riboflavin slightly above RDA. In this study, when riboflavin intake reached 10.50 mg/d, the reduced risk of coronary heart disease reached a plateau, which may be because that the conversion of riboflavin into active FAD and FMN is limited (44). This suggested that people at high risk of CHD can appropriately consume riboflavin more than RDA.

The finding in this study that riboflavin had a protective effect on CHD was consistent with some studies. Li et al. confirmed that riboflavin intake was inversely linked to all-cause mortality and cardiovascular disease mortality using the data from 10,480 adults, and the association was influenced by folate consumption (24). Another report from rat experiment revealed that a lack of riboflavin can cause cardiovascular illness by increasing levels of cardiac biomarkers and decreasing mitochondrial membrane potential (45). A case–control study compared biochemical markers of 30 patients having acute myocardial infarction with 30 healthy people, and found that riboflavin kinase levels were substantial increased in patients with acute myocardial infarction (22). Folate appeared to be the most important determinant of Hcy metabolism, and has been demonstrated to reduce Hcy levels by about 25% in the general population (46). In McNulty’s paper, riboflavin supplementation was found to dramatically lower plasma Hcy by 22% in individuals with the TT genotype and by up to 40% in those with the lowest riboflavin status at baseline (47). Riboflavin’s effect in lowering Hcy was also established in 197 premature cardiovascular disease patients by lowering blood pressure, particularly in TT genotype individuals (48). Our findings were also consistent with previous studies on B vitamins and cardiovascular health, which revealed that a higher intake of B vitamins had a protective impact against on CHD (15, 16, 49).

This study has a number of benefits. First off, given the huge sample size used, it was sufficient to produce a trustworthy result and accurate statistical power. Second, we used RCS analysis in our work to show that the dietary riboflavin intake and CHD have nonlinear connections. The trends of RCS curves and cutoff values may offer new information to health policy makers. Thirdly, we conducted subgroup analyses and interactions, took into account and adjusted for known potential risk factors for CHD, and produced more compelling results.

Nonetheless, it is also necessary to clarify a few of this study’s drawbacks. It is difficult to conclude about causation from cross-sectional data because the order of exposure and consequence could not be determined. Additionally, memory bias may have been introduced by using recall questionnaires to collect data on total dietary element intakes and CHD outcomes. Finally, we were unable to completely exclude the possibility that the observed associations were the result of unmeasured confounding variables.

## 5 Conclusion

In conclusion, our findings implied that people who consumed more riboflavin had a lower risk of CHD. In particular, among the population of older, non-smokers, non-diabetics, those with hypertension and hypercholesterolemia, or those with a BMI of 25–30 kg/m^2^, CHD risk was more closely related to riboflavin intake. Gender, drinking status and serum folate concentration might be an interaction factor influencing the effect of riboflavin intake on CHD risk. Given the global rise in the prevalence of CHD, this hypothesis might pave the way for future interventional research to investigate the influence of riboflavin intake on CHD.

## Data Availability

The datasets presented in this study can be found in online repositories. The names of the repository/repositories and accession number(s) can be found in the article/Supplementary material.
