# Supplementary material for: Associations of dietary riboflavin intake with coronary heart disease in US adults: a cross-sectional study of NHANES 2007–2018
Source: Front Nutr. 2024 Dec 12;11:1467889. doi: 10.3389/fnut.2024.1467889 (PMC11670662; doi:10.3389/fnut.2024.1467889)
Supplement: Supplementary file 1 [file Table_1.docx]

**Table S1 Detailed process of subjects exclusion**

| Step | The number of subjects before exclusion | The reasons of exclusion | NO.(subjects) | The number of subjects after exclusion |
| --- | --- | --- | --- | --- |
| 1 | 59842 | Age ＜18 | 23262 | 36580 |
| 2 | 36580 | pregnant or lactating women | 580 | 36000 |
| 3 | 36000 | inappropriate energy intake (＜ 500 or [≥](https://baike.baidu.com/item/%E2%89%A5?fromModule=lemma_inlink) 5000 kcal/day for females, and ＜ 500 or [≥](https://baike.baidu.com/item/%E2%89%A5?fromModule=lemma_inlink) 8000 kcal/day for males) | 4296 | 31704 |
| 4 | 31704 | missing coronary heart disease data (who lack information on the diagnosis of coronary heart disease) | 1741 | 29963 |
| 5 | 29963 | missing dietary data (participants with incomplete or unreliable 24-h recall dietary data) | 1889 | 28074 |
| 6 | 28074 | missing covariates data | 7349 | 20725 |
