# Supplementary material for: Associations of dietary riboflavin intake with coronary heart disease in US adults: a cross-sectional study of NHANES 2007–2018
Source: Front Nutr. 2024 Dec 12;11:1467889. doi: 10.3389/fnut.2024.1467889 (PMC11670662; doi:10.3389/fnut.2024.1467889)
Supplement: Supplementary file 2 [file Table_2.docx]

**Table S2.** **The classifications of covariates**

| Covariates | Classification |
| --- | --- |
| Age | 18-39; 40-59; ≥60 |
| Gender | Male; Female |
| Race | Mexican American; Other Hispanic; Non-Hispanic White; Non-Hispanic Black; Other Race |
| Marital status | Married/living with partner; Widowed/divorced/separated; Never married |
| Education level | Below high school; High school; Above high school |
| Poverty-income ratio (PTR) | ＜1.00; and ≥1.00 |
| Body mass index (BMI) | ≤25kg/m^2^; 25-30kg/m^2^; ≥30kg/m^2^ |
| Work activity | Vigorous; Moderate; Other |
| Recreational activity | Vigorous; Moderate; Other |
| Drinking status^a^ | Yes; No |
| Smoking status^b^ | Yes; No |
| Sleeping disorder^c^ | Yes; No |
| Hypertension^d^ | Yes; No |
| Diabetes^e^ | Yes; No |
| Hypercholesterolemia^f^ | Yes; No |
| Serum folate concentration | Continuous |
| Energy intake^g^ | Continuous |

^a^ Drinking status was stratified according to whether they have drunk at least 12 times last year. ^b^ Smoking status was stratified according to whether they smoked at least 100 cigarettes in life.^c^ Sleeping disorder was diagnosed by the question of “Ever told by doctor have sleep disorder” in questionnaire? ^d^ Systolic blood pressure (SBP) ≥130 mm Hg, or diastolic blood pressure (DBP) ≥80 mm Hg, or currently taking antihypertensive drugs were hypertensive patients. ^e^Diabetes was defined as blood glycohemoglobin ≥ 6.5%, or fasting plasma glucose ≥ 126 mg/dL, or self-reported doctor-diagnosed, or insulin use. ^f^ Hypercholesterolemia was defined as self-reported physician diagnosis or taking cholesterol-lowering drugs.^g^ Energy intake of each participant was obtained by summing the mean of the two 24h dietary intakes and the mean of two 24h intakes from supplements.
